# Endoscopic esophageal bougienage followed by modified intralesional steroid injection for refractory benign esophageal stricture: A retrospective study and meta–analysis

**DOI:** 10.1016/j.clinsp.2026.101063

**Published:** 2026-09-03

**Authors:** Yuan Tian, Yun–Long Cai, Wei–Dong Nian, Jin–Yu Liang, Wen–Zhu Li, Long Rong

**Affiliations:** Department of Endoscopy Center, Peking University First Hospital, Peking, China

**Keywords:** Refractory benign esophageal strictures, Endoscopic esophageal bougienage, Intralesional steroid injections, Retrospective study, Meta–analysis

## Abstract

**Purpose:**

To evaluate the efficacy and safety of a modified intralesional steroid injection technique following bougienage in patients with Refractory Benign Esophageal Strictures (RBES).

**Methods:**

Thirty patients with RBES were retrospectively enrolled at Peking University First Hospital between January 2012 and December 2021. All patients were treated with either endoscopic esophageal bougienage followed by modified intralesional steroid injections (using a 3 mm needle to target the submucosa at the edges of the dilation–induced mucosal tear) (n = 11) or endoscopic esophageal bougienage alone (n = 19).

**Results:**

There were no significant differences in the time to first repeat dilation between the combined therapy and bougienage–alone groups (Hazard Ratio [HR = 0.27], 95% CI 0.03 to 2.22; p = 0.189). Moreover, the authors the authors did not find a significant difference between the groups in the number of dilation sessions required (p = 0.107). In contrast, combined therapy was associated with a significantly shorter overall duration of dilation therapy (p = 0.043) and improved dysphagia severity (p = 0.044). Finally, there were no significant differences in the risks of delayed hemorrhage (p = 0.231) and perforation (p = 0.231) between the groups. Additionally, a meta–analysis of six existing studies confirmed that the combined approach was associated with significantly fewer required dilation sessions (p = 0.005) and a higher rate of dysphagia resolution (p = 0.021).

**Conclusions:**

In this small, likely underpowered, retrospective cohort, modified combined therapy did not significantly prolong the time to first repeat dilation. Observations of a shorter total duration of dilation therapy and improved dysphagia severity are considered exploratory and hypothesis–generating. The pooled evidence from our meta–analysis supports the rationale for further investigation of steroid augmentation.

## Highlights

• Bougienage with steroid injection shortens overall therapy duration in RBES.
• Combined therapy improves dysphagia severity compared to dilation alone.
• No significant difference in time to first repeat dilation between groups.
• Steroid injection shows a comparable safety profile to bougienage alone.

## Introduction

Benign Esophageal Stricture (BES) is a narrowing of the esophageal lumen caused by scar formation and fibrosis, and its main symptoms include dysphagia, food impaction, nausea, and vomiting.^1^ The risk factors for BES include anastomosis, Endoscopic Submucosal Dissection (ESD), gastroesophageal reflux, caustic injury, and radiotherapy injury.^2^ Simple strictures can often be resolved with one to three endoscopic dilations, whereas Refractory BES (RBES) requires more than three repeated endoscopic dilations, and the lumen width does not reach 14 mm after three dilation sessions.^3^ Regular endoscopic dilations are widely used for RBES; however, strictures recur within days or weeks, requiring repeat procedures, which significantly impact patients' quality of life and impose a substantial healthcare burden.

Chronic inflammation is a key factor in the development of RBES. Inflammatory processes, such as edema and muscular spasms, contribute to the initial constriction of the esophageal wall. Additionally, chronic inflammation can lead to erosions and ulcerations, which stimulate the production of fibrous tissue and collagen deposition. These collagen deposits contribute to the narrowing of the esophageal lumen.^4^ Given that approximately 30%‒40% of patients experience persistent difficulty swallowing within one year after undergoing endoscopic dilation, and considering the ability of steroids to mitigate chronic inflammation, it is reasonable to regard them as a viable therapeutic option for conditions characterized by scar formation.^4^

Conventional intralesional steroid injection, typically administered in the four quadrants of the stricture ring after dilation, has shown inconsistent outcomes, potentially due to suboptimal drug delivery to the primary site of fibrotic activity: the fresh mucosal tear.^5^ This underscores the need for a more targeted and anatomically rational injection approach. Our modified technique, which involves precise submucosal injection at the margins of the dilation–induced mucosal tear using a shorter needle, is designed to maximize steroid delivery to the site of active wound healing and fibrosis while potentially enhancing safety by minimizing deep tissue injury. Thus, this study aimed to assess the efficacy and safety of this modified technique of endoscopic esophageal bougienage followed by modified intralesional steroid injection in patients with RBES and to integrate our findings with existing evidence through a meta–analysis.

## Methods

### Patient population

This retrospective cohort study is reported in accordance with the Strengthening the Reporting of Observational Studies in Epidemiology (STROBE) statement for cohort studies. The authors comprehensively reviewed all consecutive adult patients diagnosed with RBES who underwent endoscopic bougienage at our hospital between January 2012 and December 2021. This study was approved by the Peking University First Hospital Biomedical Research Ethics Committee (n° 2022–257) and conducted in accordance with the Declaration of Helsinki. All patients were informed of the study’s purpose. The diagnosis of RBES was based on persistent dysphagia and endoscopic luminal narrowing that failed to achieve a diameter of 14 mm despite at least three previous dilation sessions.^3^ Patient allocation to the intervention or control group was determined by clinical practice at the time of the procedure. The modified steroid injection protocol was formally introduced in our practice in late 2017. Therefore, patients treated from 2012 to mid–2017 predominantly received bougienage alone (control group). From late 2017 onwards, combined therapy (bougienage plus modified steroid injection) became the preferred approach for suitable candidates, constituting the intervention group. The treating endoscopist made the final decision, based on consensus regarding the technique's availability and an assessment of individual patient factors. From the identified cohort, the authors applied the following inclusion criteria: 1) Adult patients aged 18.0 years or older; 2) BES including esophageal stricture after esophagectomy or extensive ESD surgery; 3) Patients treated with dilation bi–weekly; and 4) Dysphagia and endoscopic failure that persisted at the endoscopic re–examination two weeks after the third dilation. Patients with any of the following were excluded: 1) Contraindications for endoscopic treatment, including coagulation dysfunction (INR >1.5, platelet count <50 × 10^9^/L), severe hepatic (Child–Pugh class C) or renal dysfunction (Egfr <30 mL/min/1.73 m^2^), and unstable cardiopulmonary dysfunction; 2) patients with multifocal or diffuse esophageal mucosal lesions; 3) patients with esophagotracheal fistula and esophagomediastinal fistula; 4) patients receiving oral steroids or esophageal stents; and 5) patients who did not sign the informed consent form.

**Fig. 1:**
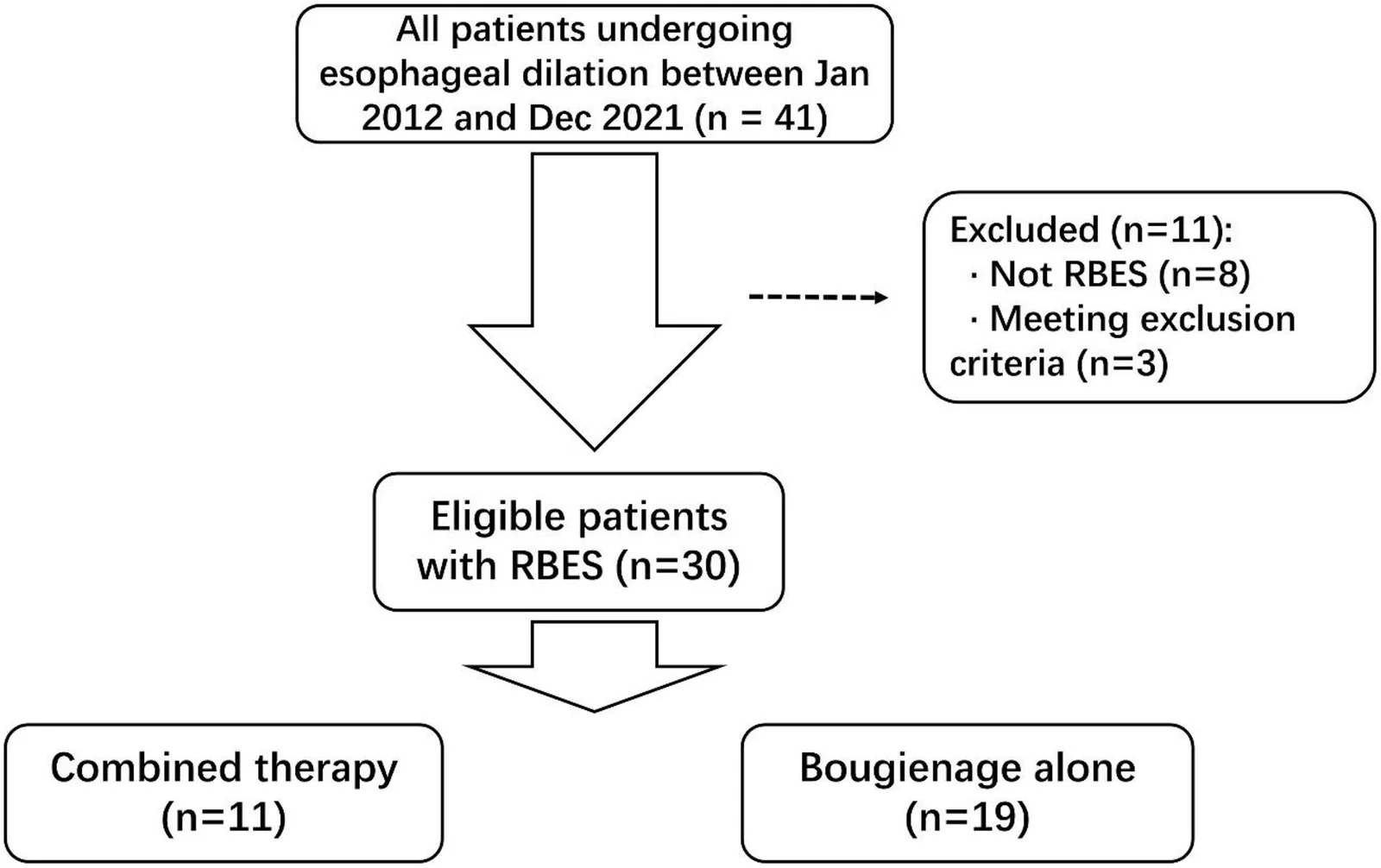
Flow diagram of patient selection. RBES, Refractory Benign Esophageal Stricture.

The screening and inclusion processes are summarized in Fig. 1. The initial database search identified 41 patients who underwent esophageal dilation. After applying the RBES definition and the study's inclusion/exclusion criteria, 30 eligible patients were included in the final analysis, representing all consecutive eligible RBES cases managed with bougienage during the study period at our institution.

### Intervention and control

**Fig. 2:**
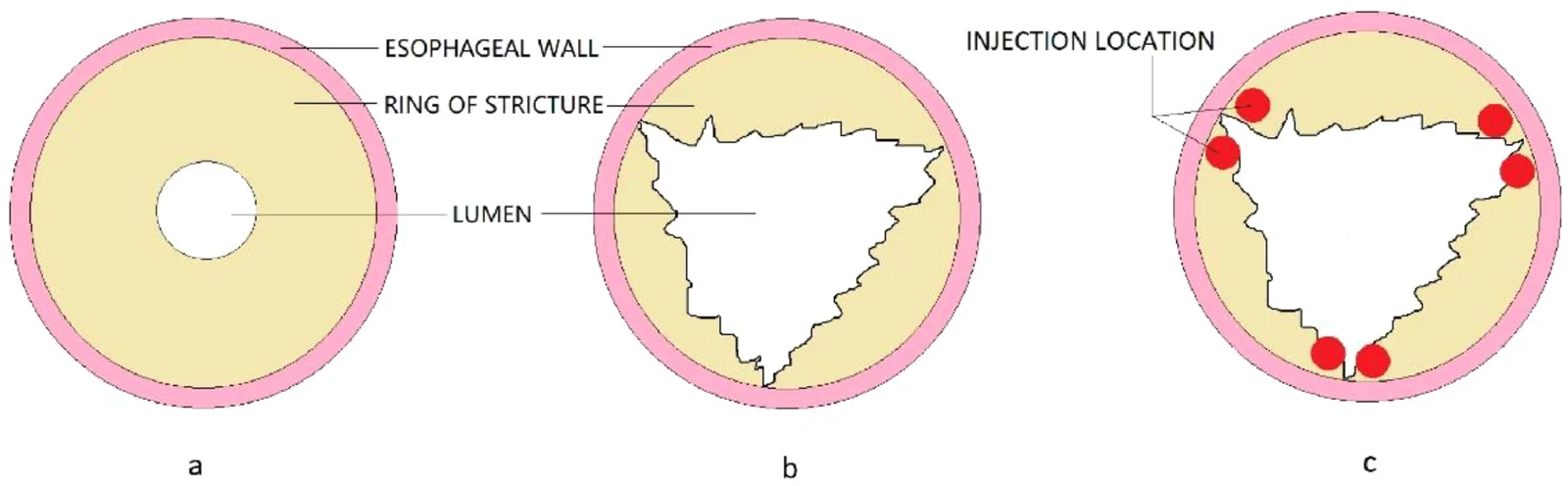
Injection location. (a) Benign esophageal stricture. (b) The ring structure was torn after the extension of the bougienage. (c) Steroid was injected around the wound.

All endoscopic procedures were performed under conscious sedation by experienced endoscopists. In the intervention group, patients underwent bougienage dilation using Savary–Gilliard dilators (Cook Medical, USA) up to a diameter of 15 mm under endoscopic and fluoroscopic guidance. The dilation endpoint was defined as the passage of the 15 mm dilator with moderate resistance. Immediately after dilation, endoscopic injection was performed using a 3 mm sclerotherapy needle (Interject®, Boston Scientific, USA). Triamcinolone acetonide (Specification: 1 mL: 40 mg) was diluted with 0.9% NaCl to a concentration of 10 mg/mL. Using the 3–mm needle, multiple submucosal injections (0.5 mL per injection point, containing 5 mg of triamcinolone) were administered meticulously at the periphery and base of the fresh mucosal tear created by bougienage. The injection was performed from the distal edge of the wound, proceeding to the proximal edge, ensuring even distribution on both sides of the laceration (Fig. 2). Prior to steroid injection, a preliminary test with a small amount of normal saline was performed to confirm submucosal placement, evidenced by the formation of a bleb. This step aimed to ensure accurate localization and avoid inadvertent injection into the muscularis propria or deeper layers. The total dose of triamcinolone acetonide was kept within 80 mg per session. Patients in the control group were treated with bougienage dilation alone using the same protocol (Savary–Gilliard dilators up to 15 mm). The procedural endpoint was identical: passage of the 15–mm dilator with moderate resistance. Post–dilation, no sham injections were performed.

### Data collection and outcome measurement

**Fig. 3:**
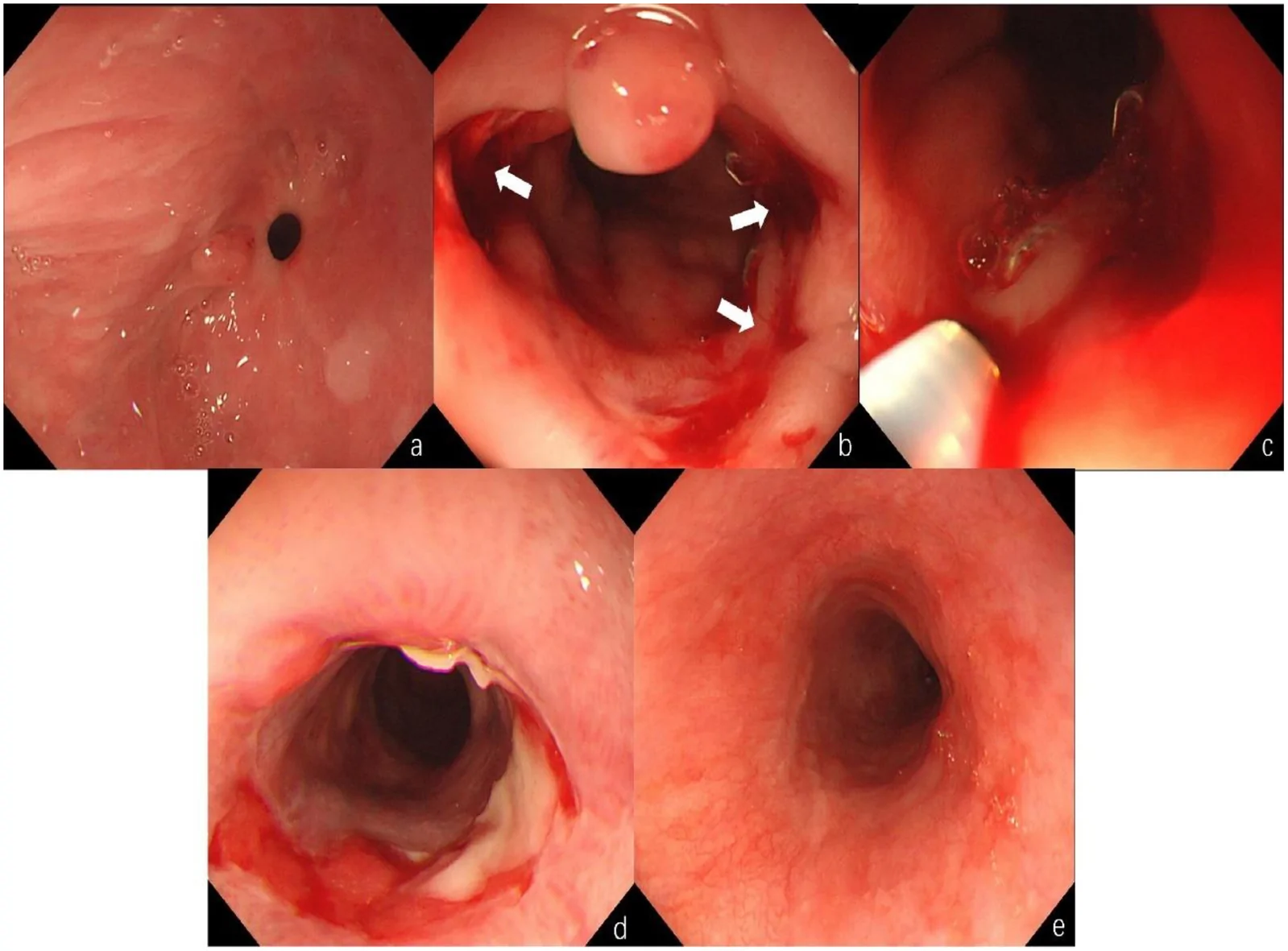
Typical endoscopic views of the esophagus in a patient in the intervention group. (a) Benign esophageal stricture after esophagectomy. (b) The laceration wound (white arrow) after treatment with 15 mm bougienage dilation. (c) Triamcinolone was injected using a 3 mm injection needle at the edge of the mucosal tear wound. (d) Ulcer around the stricture at the 2–week follow–up after the injection. (e) The stenosis was relieved at the 1–month follow–up.

General information was collected, including sex (male/female), age (years), stricture location (cervical, middle, distal, or double), etiology of strictures (surgery, after ESD, and others), severity of dysphagia (graded using the Stooler scale), and follow–up duration. The patients were followed up for postoperative dysphagia symptoms at 2 weeks and at 1–, 3–, and 6–months, and the degree of esophageal stricture and lumen diameter were evaluated by gastroscopy (Fig. 3). The decision to apply dilation treatment was based on the presence of dysphagia symptoms. If dilation was deemed necessary, the intervention group underwent repeated endoscopic esophageal bougienage followed by intralesional steroid injection, while the control group underwent repeated endoscopic esophageal bougienage alone. Follow–up was continued if no further dilation was clinically indicated. If the patient had no obvious dysphagia (Stooler grade II or lower) and the stenosis could be passed by endoscopy at the 6–month follow–up, the stenosis was considered relieved, and the patient was followed up for at least 1–year after treatment. If dysphagia worsened during the follow–up period, patients were instructed to visit the doctor at any time to review the gastroscopy and repeat the above dilation treatment. The primary endpoint was the time to first repeat dilation, while the secondary endpoints included the number of required dilation sessions, duration of dilation therapy, dysphagia severity, and complications.

### Statistical analysis

Given the retrospective, exploratory nature of this study and the challenge of predefining effect sizes for a novel technique in a refractory population, a formal a priori sample–size or power calculation was not performed. The sample size was determined by the available cohort meeting the inclusion criteria during the study period. The variables collected in the intervention and control groups were treated as continuous and categorical data. Continuous variables are reported as mean (standard deviation) or median (interquartile range) based on data distribution, while categorical variables are presented as events (frequencies). The log–rank test was used to assess the time to first repeat dilation, and differences between the groups were evaluated using the Kaplan–Meier method. The chi–square and independent *t*–tests were used to compare categorical and continuous outcomes, respectively. Statistical significance was defined as a two–tailed p < 0.05. Statistical analyses were performed using SPSS version 24.0 for Windows.

### Meta–analysis

To integrate these findings with existing evidence, the authors conducted a systematic review and meta–analysis in accordance with recommended guidelines. This component is reported in accordance with the Preferred Reporting Items for Systematic Reviews and Meta–Analyses (PRISMA) statement. A comprehensive systematic literature search was conducted in the following electronic databases from their inception until September 2025: PubMed, Embase (via Ovid), and the Cochrane Central Register of Controlled Trials (CENTRAL). The search strategy was designed in consultation with a medical librarian and utilized a combination of Medical Subject Headings (MeSH) and free–text terms related to esophageal strictures and steroid injections. The full search strategy for PubMed is provided as a Supplementary File (Supplementary File 1); analogous strategies were adapted for other databases. The reference lists of all retrieved articles and relevant review articles were manually screened to identify additional studies. No language restrictions were applied initially.

Two investigators (initials blinded) independently screened the titles and abstracts of all identified records. Full–text articles of potentially eligible studies were then retrieved and independently assessed by the same two investigators against the predefined eligibility criteria. Any discrepancies at any stage were resolved through discussion or by consulting a third senior investigator. The authors The authors included studies that met the following PICOS criteria: 1) Patients: adults with RBES; 2) Intervention: endoscopic dilation combined with intralesional steroid injection; 3) Comparison: endoscopic dilation alone; 4) Outcomes: time to first repeat dilation, number of required dilation sessions, duration of dilation therapy, dysphagia severity, and complications; and 5) Study design: Randomized Controlled Trials (RCTs) or comparative cohort studies (prospective or retrospective).

Data extraction was performed using a standardized form that captured study characteristics (first author, publication year, country, study design), sample size, stricture etiology, previous dilation, intervention details, and outcomes of interest. The risk of bias for the included studies was assessed using the Newcastle–Ottawa Scale (NOS).^6^ The pooled effect estimates are reported as WMD and OR with 95% Confidence Intervals for continuous and categorical data, and the pooled analyses were calculated using the random–effects model, which could consider the underlying heterogeneity across included studies.^7^ Heterogeneity among the included studies was then assessed using *I*^2^ and *Q* statistics, and *I*^2^ ≥ 50% or p < 0.10 was considered significant heterogeneity.^8^^,^^9^ All reported p–values are two–sided, and p < 0.05 was considered statistically significant. The meta–analysis was performed using Stata.

## Results

### Baseline characteristics

Of the 30 included patients, 11 were treated with endoscopic esophageal bougienage followed by intralesional steroid injection, whereas the remaining 19 patients were treated with endoscopic esophageal bougienage alone. Regarding the etiology of strictures, as detailed in Table 1, the majority of cases in both groups were postoperative: anastomotic strictures after surgery (20/30, 66.7%) and strictures following extensive Endoscopic Submucosal Dissection (ESD) (8/30, 26.7%). The remaining two cases were attributed to caustic ingestion and severe reflux esophagitis. Notably, no cases of radiation–induced stricture were included in this cohort. The predominance of postoperative strictures, particularly anastomotic strictures, reflects the major causes of RBES in our tertiary referral center. The homogeneous etiology, predominantly fibrotic postsurgical strictures, provides a focused context for interpreting the therapeutic outcomes. However, it also limits the generalizability of our findings to other etiologies such as radiation or peptic strictures. Moreover, there were no significant differences between the intervention and control groups for sex (p = 0.559), age (p = 0.681), stricture location (p = 0.241), etiology (p = 0.691), dysphagia severity (p = 0.619), or follow–up duration (p = 0.152).

**Table 1:** The baseline characteristics of included patients.

Variables	Intervention group (n = 11)	Control group (n = 19)	p–value
Sex (%)			
Male	9	17	0.559
Female	2	2	
Age (years)	67.00±9.85	65.53±9.10	0.681
Stricture location			
Cervical	8	7	0.241
Middle	1	3	
Distal	2	8	
Double	0	1	
Etiology of strictures			
Surgery	8	12	0.691
ESD	2	6	
Other*	1	1	
Dysphagia severity			
Normal	0	0	0.619
Mild	0	0	
Moderate	9	16	
Severe	2	3	
Follow–up period (months)	44.50±28.61	60.90±29.82	0.152

^a^Including Chemical burn stricture, severe reflux esophagitis stricture.

### Investigated endpoints

**Fig. 4:**
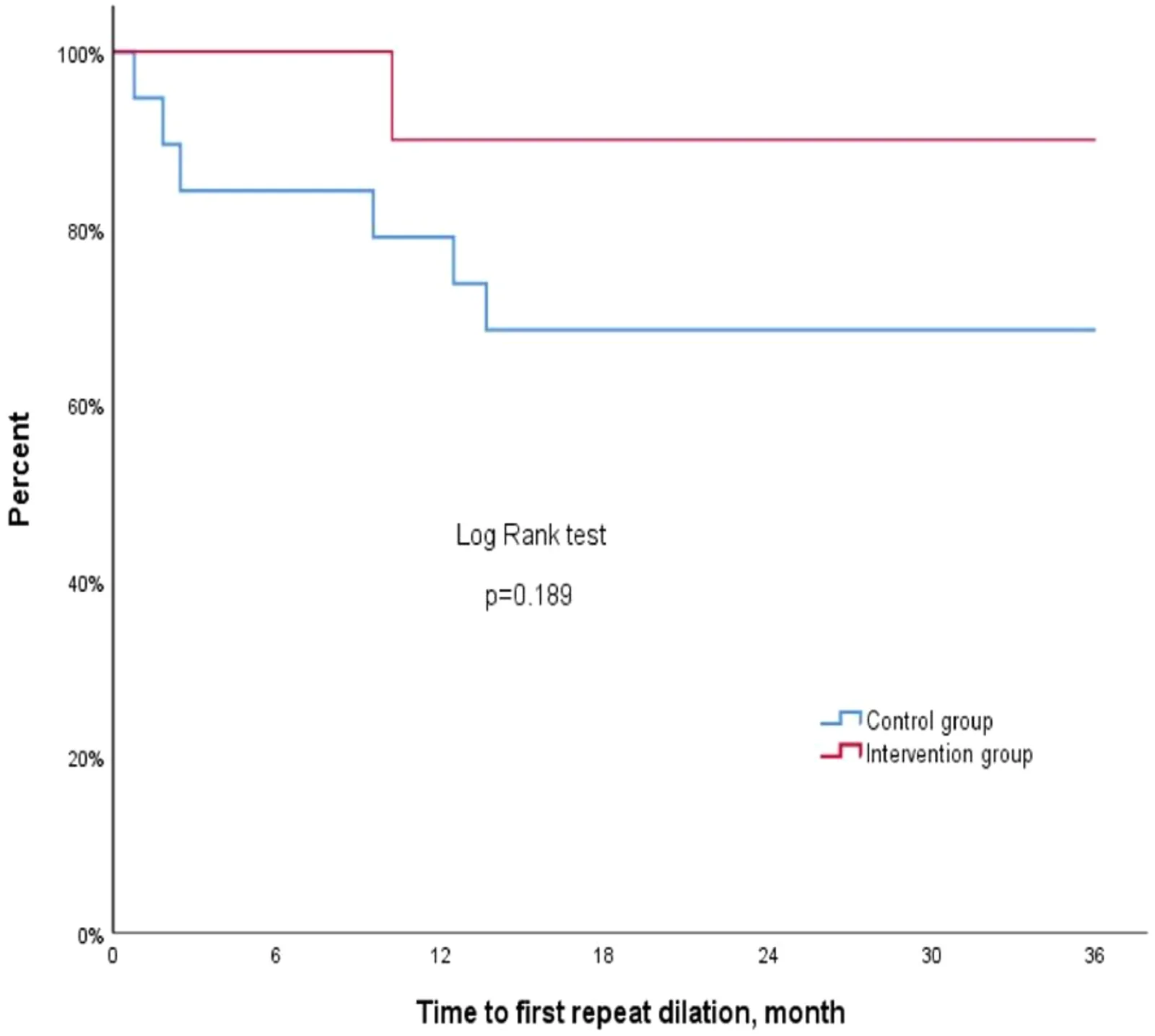
Kaplan–Meier curve for the time to first repeat dilation in the intervention and control groups.

The time to first repeat dilation between the intervention and control groups is shown in Fig. 4. A univariable Cox regression analysis showed that the combined therapy was associated with a numerically lower hazard for requiring repeat dilation compared to bougienage alone, although this difference was not statistically significant (Hazard Ratio [HR = 0.27], 95% CI 0.03 to 2.22; p = 0.189). Moreover, the use of endoscopic esophageal bougienage followed by intralesional steroid injection was not associated with the number of required dilation sessions compared with endoscopic esophageal bougienage alone (mean difference: −3.38; 95% CI −7.59 to 0.83; p = 0.107; Table 2). Furthermore, the duration of dilation therapy was significantly shorter in the intervention group than in the control group (mean difference: −3.84; 95% CI −7.55 to −0.13; p = 0.043; Table 2). Additionally, the authors the authors noted that endoscopic esophageal bougienage followed by intralesional steroid injection was associated with a greater improvement in the severity of dysphagia compared to endoscopic esophageal bougienage alone (p = 0.044; Table 2). Finally, there were no significant differences between the intervention and control groups in the risk of delayed hemorrhage (p = 0.231) or perforation (p = 0.231).

**Table 2:** Investigated outcomes.

**Variables**	**Intervention group (n=11)**	**Control group (n=19)**	**p–value**
Required dilatation sessions	6.36±1.69	9.74±6.54	0.107
Duration of dilatation therapy (months)	3.41±1.20	7.25±5.90	0.043
Dysphagia severity			
Normal	8	5	0.044
Mild	2	9	
Moderate	1	5	
Severe	0	0	
Complication			
Delayed hemorrhage	0	1	0.231
Perforation	0	1	0.231

### Meta–analysis

**Fig. 5:**
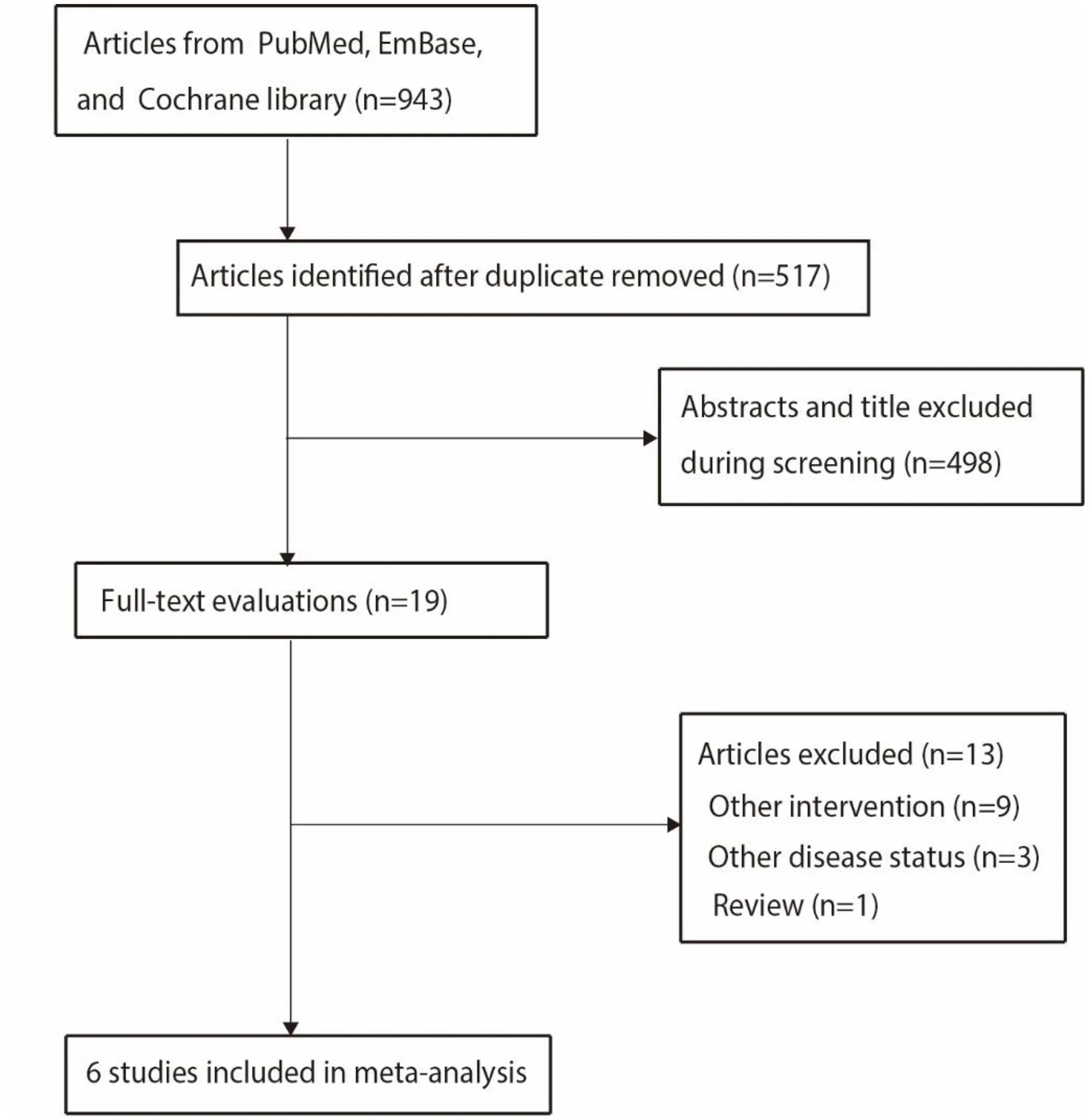
PRISMA flow diagram of the systematic review process for the meta–analysis.

**Fig. 6:**
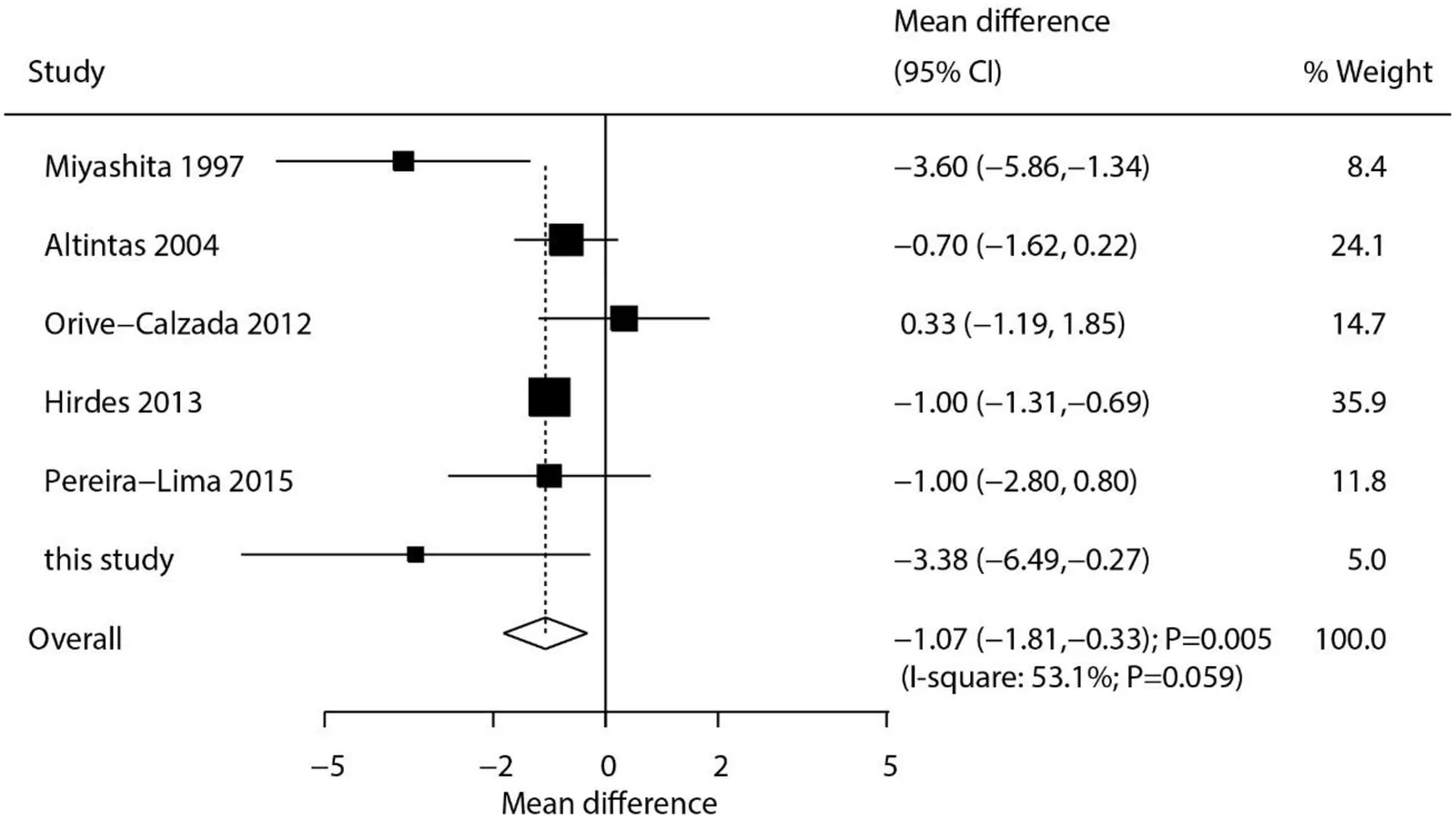
Effect of endoscopic esophageal bougienage plus intralesional steroid injection on the required dilation sessions.

**Fig. 7:**
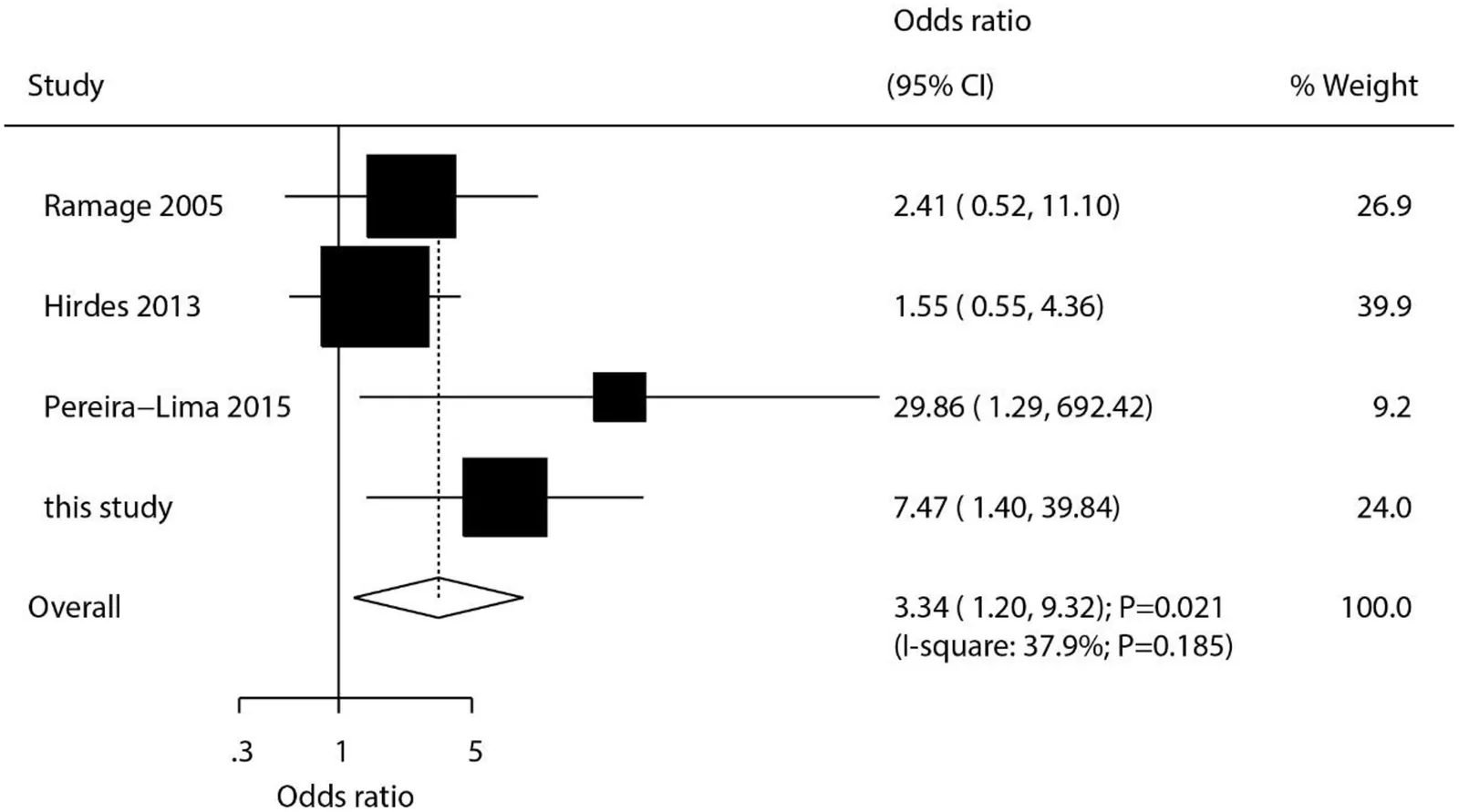
Effect of endoscopic esophageal bougienage plus intralesional steroid injection on the incidence of absence of dysphagia.

The study selection process is summarized in the PRISMA flow diagram (Fig. 5). A total of six studies were identified from electronic searches and selected for the final meta–analysis.^10^, ^11^, ^12^, ^13^, ^14^, ^15^ The baseline characteristics of the identified studies and involved patients are shown in Table 3. The risk of bias assessment indicated that the cohort studies had NOS scores of 4‒6, indicating moderate quality. The authors The authors noted that endoscopic esophageal bougienage plus intralesional steroid injection was associated with fewer required dilation sessions (WMD: −1.07; 95% CI −1.81 to −0.33; p = 0.005; *I*² = 53.1%, p for heterogeneity = 0.059; Fig. 6) and a higher incidence of absence of dysphagia (OR = 3.34; 95% CI 1.20 to 9.32; p = 0.021; *I*² = 37.9%, p for heterogeneity = 0.185; Fig. 7).

**Table 3:** Baseline characteristics of identified studies and involved patients.

**Study**	**Country**	**Sample size**	**Stricture etiology**	**Previous dilation**	**Intervention**	**Control**	**NOS**
Miyashita 1997.^8^	Japan	33	Anastomotic: 33	No	TTS–CRE, 2 mg dexamethasone into each quadrant at the anastomosis after dilation	SGBD or TTS–CRE	5
Altintas 2004.^9^	Turkey	21	Anastomotic:4; corrosive: 3; peptic: 10; radiation induced: 4	Yes	SGBD, 8 mg triamcinolone into each quadrant after dilation	SGBD	5
Ramage 2005.^10^	USA	30	Peptic: 30	Yes	TTS–CRE, 20 mg triamcinolone into each quadrant of the narrowest region of the stricture before dilation	TTS–CRE and sham injection	5
Orive–Calzada 2012.^11^	Spain	23	Anastomotic:4; corrosive: 6; peptic: 12; radiation induced: 1	No	SGBD or TTS–CRE, 20 mg triamcinolone into each quadrant before dilation	SGBD or TTS–CRE	4
Hirdes 2013.^12^	Netherlands	60	Anastomotic: 60	No	SGBD, 20 mg triamcinolone into each quadrant before dilation	SGBD and sham injection	6
Pereira–Lima 2015.^13^	Brazil	19	Anastomotic: 19	No	SGBD, 40 mg triamcinolone at the border of the mucosal tears caused by dilation after dilation	SGBD and sham injection	5
This study	China	30	Anastomotic: 30	Yes	SGBD, followed by modified submucosal triamcinolone injection (≤80 mg) at the edges of the dilation–induced mucosal tear using a 3–mm needle	SGBD	5

SGBD, Savary–Gilliard Bougie Dilation; TTS–CRE, Through–The–Scope Controlled Radial Expansion balloon dilation.

## Discussion

The occurrence rate of esophageal anastomotic stricture, which is the most common complication of esophageal cancer surgery, ranges from 5.0% to 46.0%.^16^ The frequency of postoperative esophageal strictures has been on the rise owing to the widespread use of ESD. When lesions occupy between 1/2 and 3/4 of the periesophageal circumference, the incidence of esophageal stenosis is approximately 20%. However, when lesions occupy >3/4 of the periesophageal circumference and undergo endoscopic resection, the incidence of esophageal stenosis ranges from 83.3% to 94.1%.^17^ Postoperative esophageal stenosis is a type of fibrotic narrowing, and symptoms of obstruction can be relieved using a probe and balloon to achieve dilation. However, the majority of patients do not respond well to treatment and experience recurrent stenosis, necessitating repeated dilation procedures. In this study, the therapeutic effects of a modified endoscopic esophageal bougienage technique followed by intralesional steroid injection were assessed in patients with RBES. Thirty patients with a broad range of characteristics were identified. The authors noted that endoscopic esophageal bougienage followed by intralesional steroid injection was associated with a shorter duration of dilation therapy and improved dysphagia severity compared with endoscopic esophageal bougienage alone. However, there were no significant differences between the groups in time to first repeat dilation, number of required dilation sessions, delayed hemorrhage, and perforation. The meta–analysis of the existing literature corroborated our key findings, demonstrating that the combined approach significantly reduced the number of dilation sessions needed and increased the likelihood of dysphagia resolution.

Currently, balloon and bougienage dilation is considered the first–line treatment for RBES, and combining this treatment with steroids could improve postoperative esophageal strictures by reducing the number of dilation sessions and prolonging the relief time of dysphagia.^4^^,^^18^ However, several other studies have reported inconsistent results in patients with RBES.^14^^,^^15^ For example, Hirdes et al. conducted a multicenter, double–blind trial and identified 60 patients with untreated cervical anastomotic stricture after esophagectomy with gastric tube reconstruction and dysphagia. They found that four–quadrant injections of 0.5 mL triamcinolone into the stricture, followed by Savary dilation to 16 mm, did not improve the frequency of repeat dilation sessions or the dysphagia–free period.^14^ In contrast, Pereira–Lima et al. identified 19 patients with non–dilated esophagogastric complex strictures after esophagectomy with gastric pull–up and found that injection of triamcinolone after dilation at every session significantly improved dysphagia.^15^ One possible explanation for the varying outcomes could be the administration of triamcinolone injections at different stages.

The present findings should be interpreted in the context of previous systematic reviews and meta–analyses on this topic, which have highlighted both potential benefits and significant uncertainty. Szapáry et al., in a meta–analysis of 11 studies, reported that intralesional steroid injection significantly increased the time between dilations (improved periodic dilation index) but did not significantly reduce the total number of repeat dilations or improve dysphagia scores, noting substantial heterogeneity.^19^ Similarly, a systematic review by Henskens et al. concluded that evidence for the efficacy of steroid injections was of very low certainty and highly etiology–dependent, showing benefits for peptic, radiation–induced, and corrosive strictures but inconsistent results for the common subgroup of anastomotic strictures.^20^ This study, which primarily included postoperative strictures (anastomotic and post–ESD), adds to this nuanced landscape. While our single–center retrospective data did not show a significant prolongation of the time to first repeat dilation, the authors observed a significant reduction in the total duration of active dilation therapy and an improvement in dysphagia severity.

Furthermore, our meta–analysis, which incorporated more recent studies, found a significant reduction in the number of dilation sessions required. This suggests that our modified injection technique, specifically targeting the fresh wound edge, may offer a more effective delivery method, potentially mitigating the inconsistent outcomes previously reported for anastomotic strictures and contributing to the pooled evidence of benefits seen in our updated synthesis. Nevertheless, as correctly noted in these prior reviews, the evidence remains limited by study heterogeneity and design limitations, underscoring the need for high–quality trials.

The present study, utilizing a modified injection technique, observed significant improvements in therapy duration and dysphagia, potentially bridging this gap in evidence. The mechanistic rationale lies in the antifibrotic properties of steroids. Dilation–induced mucosal tears trigger an acute inflammatory response, characterized by infiltration of inflammatory cells and proliferation of fibroblasts, leading to collagen deposition and scar formation, the hallmark of recurrent strictures.^21^ Corticosteroids, such as triamcinolone, exert their effect by suppressing inflammatory cell recruitment, inhibiting fibroblast activation and collagen synthesis, and enhancing collagenase activity, thereby disrupting the fibrotic cascade.^22^ Our modified technique, which precisely targets steroid injection to the fresh wound edges created by bougienage, aims to maximize the local drug concentration at the primary site of fibrotic activity. This targeted approach may lead to a more effective suppression of inflammation and fibrosis than conventional quadrant injections into the mature stenotic ring, potentially explaining the observed clinical benefits in our cohort and offering a possible explanation for the heterogeneous results in the broader literature.

This study found that the duration of dilation therapy and dysphagia severity could improve in patients treated with endoscopic esophageal bougienage followed by intralesional steroid injections. Moreover, although differences in the time to first repeat dilation, number of required dilation sessions, delayed hemorrhage, and perforation between the groups were not statistically significant, these results could be explained by the smaller number of included patients, indicating that our conclusion is not sufficiently statistically powered and not applicable to wider populations. Notably, the trends favoring the combined therapy for time to first repeat dilation and number of sessions, coupled with the significant findings in the meta–analysis, suggest a genuine treatment effect that our single–center study might have been underpowered to definitively confirm. The meta–analysis found that endoscopic esophageal bougienage plus intralesional steroid injection was associated with fewer dilation sessions and an increased incidence of the absence of dysphagia. Several reasons may account for the improved outcomes observed in patients who underwent intralesional steroid injections. Following esophagectomy, mucosal defects and artificial ulcers may develop, accompanied by acute inflammatory reactions. In response, the surrounding normal epithelial cells regenerate, leading to hyperplasia of the submucosal granulation tissue. This process is characterized by the infiltration of inflammatory cells, angiogenesis, and proliferation of fibroblasts, ultimately resulting in scar formation during ulcer repair. These scar tissues can potentially decrease the compliance and contractility of the esophageal wall, thereby contributing to the development of esophageal strictures.^23^ Steroids can suppress the release of inflammatory cells, inhibit the activation and movement of fibroblasts and inflammatory cells, enhance collagenase activity, and decrease collagen accumulation and scar formation.^24^ Moreover, studies have shown that oral steroids can lower the incidence of esophageal stricture after esophagectomy or extensive ESD surgery and reduce the frequency of dilation procedures.^25^, ^26^, ^27^

Nevertheless, patients treated with oral steroids experienced a notable elevation in the likelihood of postoperative infection, osteoporosis, and ulcers. Despite this drawback, the use of local steroids can mitigate the risk of unfavorable outcomes due to the administration of lower doses and shorter duration of exposure. Studies have found that the use of local steroids significantly reduces both the risk of esophageal stricture after ESD and the number of required dilation sessions.^28^^,^^29^ Finally, a meta–analysis of six RCTs, four crossover trials, and one cohort study indicated that intralesional steroid injection in addition to endoscopic dilation significantly increased the time to subsequent endoscopic dilations in patients with benign refractory esophageal strictures. In contrast, the number of repeat dilations and dysphagia scores did not differ significantly between the groups.^19^ These inconsistent results could be explained by the histological activity of the inflammation of the strictures and their different etiologies.^30^, ^31^, ^32^, ^33^ The studied cohort consisted almost exclusively of postoperative strictures, which may represent a subgroup with a particularly pronounced fibrotic response, where targeted antifibrotic therapy could be most relevant. The absence of radiation–induced strictures, known for their dense fibrosis and particularly challenging course, precludes any conclusions for this subgroup.

The primary innovation of this study is the detailed description and application of the modified injection technique. By utilizing a shorter (3 mm) injection needle and directing the steroid precisely to the submucosal layer at the edges of the fresh mucosal tear post–dilation, we aimed to enhance both efficacy and safety. Theoretically, the use of a shorter needle reduces the risk of transmural injection and injury to the muscularis propria, potentially mitigating complications, such as perforation or mediastinal abscess. Saline test injection prior to steroid administration further ensures proper needle placement within the submucosa. This methodological refinement addresses a potential limitation of conventional techniques and may contribute to the favorable safety profile observed in the present study, in which complication rates were comparable between the groups.

Nowadays, local steroid injection involves the average multi–point injection of triamcinolone into the four quadrants of the stenotic ring after dilation; the concentration is typically 5‒20 mg/mL, and the total dose ≤80 mg/session.^34^^,^^35^ Several studies have found that local injection of triamcinolone at the base of wound ulcers can reduce the risk of esophageal stricture after ESD, and fibrocyte hyperplasia mainly appears at the wound site.^36^, ^37^, ^38^, ^39^ In this study, the intralesional steroid injection method was changed to multiple submucosal injections at the tear site after dilation treatment because of the risk of bleeding, perforation, and increased the risk of mediastinal abscess caused by local steroid injection into the muscularis propria or through the esophageal wall.^40^ The most commonly used submucosal injection needle is 4 mm in length, and the full thickness of the esophageal wall is easily penetrated during injection. Therefore, this study used a 3 mm injection needle to prevent injection into the muscularis propria at the wound site.

This study had several important limitations. First, its retrospective design may have introduced selection and information biases. Most critically, the small sample size, coupled with the absence of a prior power calculation, suggests that this study was likely underpowered, particularly for the primary endpoint of time to first repeat dilation. Therefore, the non–significant result for this endpoint should not be interpreted as evidence of no difference, and the significant findings for secondary outcomes (duration of therapy and dysphagia score) must be viewed as preliminary and hypothesis–generating rather than definitive. These factors limit the generalizability of our single–center findings. Finally, we lacked data on inflammatory biomarkers, which could have provided mechanistic insights.

## Conclusion

In this exploratory, retrospective study, the described modified technique of intralesional steroid injection following bougienage was associated with a shorter duration of dilation therapy and improved dysphagia severity compared to bougienage alone. However, the study was not powered to detect a difference in the primary endpoint. These findings should be interpreted as preliminary and hypothesis–generating. The pooled evidence from our meta–analysis corroborates the potential benefit of steroid injections in reducing the number of dilation sessions. The modified injection technique, emphasizing targeted wound–edge injection, represents a promising refinement that merits rigorous evaluation in future, adequately powered, prospective RCTs to establish its definitive efficacy and safety.

## Statement of ethics

This study was approved by the Peking University First Hospital Biomedical Research Ethics Committee (n°2022–257) in accordance with the ethical standards of the 1964 Declaration of Helsinki and its later amendments.

## Informed consent

Written informed consent was obtained from all patients.

## Consent for publication

Not applicable.

## Author's contribution

Conception and design: RL and TY; Analysis and interpretation of the data: TY and CYL；Drafting of the article: TY. Critical revision for important intellectual content: RL and NWD; Provision of study materials or patients: CYL, LJY and LWZ; Obtaining funding: TY and RL. Collection and assembly of data: LJY and LWZ. All authors reviewed the manuscript. The author(s) read and approved the final manuscript.

## Declaration of competing interest

The authors declare no conflicts of interest.

## Data Availability

The datasets generated and analyzed during the retrospective study are not publicly available due to patient privacy and confidentiality regulations, but are available from the corresponding author upon reasonable request, subject to institutional review board approval. The data supporting the findings of the meta–analysis were derived from studies cited in the reference list.

## Appendix Supplementary materials

Image, application 1

Image, application 2

Image, application 3

Image, application 4
