# Supplementary material for: Endoscopic esophageal bougienage followed by modified intralesional steroid injection for refractory benign esophageal stricture: A retrospective study and meta–analysis
Source: Clinics (Sao Paulo). 2026 Sep 3;81:101063. doi: 10.1016/j.clinsp.2026.101063 (PMC13571524; doi:10.1016/j.clinsp.2026.101063)
Supplement: Supplementary file 3 [file mmc4.docx]

**PubMed:**

#1 "Esophageal Stenosis"[Mesh] OR "esophageal stricture"[tiab] OR "benign esophageal stricture*"[tiab] OR "refractory esophageal stricture*"[tiab] OR "esophageal stenosis"[tiab] OR "esophageal strictures"[tiab] OR ((esophageal[tiab] OR oesophageal[tiab]) AND (stricture[tiab] OR stenos*[tiab]))

#2 "Injections, Intralesional"[Mesh] OR "intralesional injection*"[tiab] OR "steroid injection*"[tiab] OR "corticosteroid injection*"[tiab] OR "triamcinolone"[tiab] OR "triamcinolone acetonide"[tiab] OR "submucosal steroid injection*"[tiab] OR "local steroid injection*"[tiab]

#3 #1 AND #2

#4 ("Animals"[Mesh] NOT "Humans"[Mesh])

#5 #3 NOT #4

**EmBase:**

1. exp esophagus stenosis/ or (esophageal stricture or benign esophageal stricture or refractory esophageal stricture or esophageal stenosis).ti,ab,kw.

2. exp intralesional drug administration/ or (intralesional injection or steroid injection or corticosteroid injection or triamcinolone or triamcinolone acetonide).ti,ab,kw.

3. 1 and 2

4. (animal/ or nonhuman/ or animal experiment/) not (human/ or human experiment/)

5. 3 not 4

**Cochrane library:**

#1 MeSH descriptor: [Esophageal Stenosis] explode all trees

#2 (esophageal stricture or benign esophageal stricture or refractory esophageal stricture):ti,ab,kw

#3 #1 or #2

#4 MeSH descriptor: [Injections, Intralesional] explode all trees

#5 (intralesional injection or steroid injection or corticosteroid injection or triamcinolone):ti,ab,kw

#6 #4 or #5

#7 #3 and #6
